# Comprehensive Analysis of *Artemisia argyi* H. Lév. & Vaniot Essential Oil: Chemical Composition, Antimicrobial Efficacy, Biofilm Disruption, and Insecticidal Potential

**DOI:** 10.1002/fsn3.71385

**Published:** 2026-06-25

**Authors:** Miroslava Kačániová, Zhaojun Ban, Li Li, Jian Lou, Joel Horacio Elizondo‐Luevano, Anis Ben Hsouna, Rania Ben Saad, Alessandro Bianchi, Ladislav Bakay, Stefania Garzoli

**Affiliations:** ^1^ Institute of Horticulture Faculty of Horticulture and Landscape Engineering Slovak University of Agriculture Nitra Slovakia; ^2^ School of Medical & Health Sciences VIZJA University Warszawa Poland; ^3^ School of Biological and Chemical Engineering Zhejiang University of Science and Technology, Zhejiang Provincial Key Laboratory of Chemical and Biological Processing Technology of Farm Products, Zhejiang Provincial Collaborative Innovation Center of Agricultural Biological Resources Biochemical Manufacturing Hangzhou China; ^4^ Key Laboratory for Agro‐Products Postharvest Handling of Ministry of Agriculture and Rural Affairs, College of Biosystems Engineering and Food Science Zhejiang University Hangzhou China; ^5^ Laboratory of Natural Sciences, Biomolecular Innovation Group, Faculty of Agronomy Universidad Autónoma de Nuevo León Cd. Gral. Escobedo Nuevo León Mexico; ^6^ Laboratory of Biotechnology and Plant Improvement, Centre of Biotechnology of Sfax Sfax Tunisia; ^7^ Department of Environmental Sciences and Nutrition, Higher Institute of Applied Sciences and Technology of Mahdia University of Monastir Monastir Tunisia; ^8^ Department of Agriculture, Food and Environment University of Pisa Pisa Italy; ^9^ Institute of Landscape Architecture, Faculty of Horticulture and Landscape Engineering Slovak University of Agriculture Nitra Slovakia; ^10^ Department of Chemistry and Technologies of Drug Sapienza University Rome Italy

**Keywords:** antimicrobial activity, *Artemisia argyi*, biofilm inhibition, essential oil, food preservation, GC–MS, insecticidal activity, MALDI‐TOF MS

## Abstract

*Artemisia argyi* is a traditional medicinal plant known for its broad spectrum of biological activities, including antimicrobial, anti‐inflammatory, and insecticidal effects. In this study, the essential oil (EO) extracted from the leaves of *A. argyi* (AAEO) was comprehensively analyzed by GC–MS to determine its chemical composition. The antimicrobial efficacy was evaluated in vitro by disc diffusion and determination of minimum inhibitory concentrations (MIC) against selected bacterial and yeast pathogens. The antimicrobial effect was tested in situ on various food matrices (apple, banana, beetroot, cucumber) by vapor phase exposure. Antibiofilm activity was evaluated by crystal violet assay and MALDI‐TOF MS analysis of biofilm samples. The insecticidal activity was evaluated against the pest *Callosobruchus maculatus* . The results showed that AAEO consists predominantly of 1,8‐cineole (20.5%), *β*‐caryophyllene (16.5%) and longifolene (13.8%). The oil showed mild to moderate antimicrobial activity, with stronger effects against Gram‐positive bacteria, and significantly inhibited microbial growth on food matrices, with efficacy depending on the type of matrix and oil concentration. AAEO also reduced the biofilm formation of *Salmonella enterica* , which was confirmed by changes in the MALDI‐TOF MS spectra of the biofilm. Insecticidal activity was significant at concentrations of 25% and above, with 100% mortality achieved at a concentration of 100%. These results support the potential of AAEO as a natural bioactive agent suitable for food safety, crop protection and integrated pest management applications.

## Graphical abstract

Comprehensive analyses, including GC–MS profiling, antimicrobial and antibiofilm assays, vapor‐phase application, and insecticidal evaluation, reveal the strong multifunctional potential for practical use in food and agricultural protection of *Artemisia argyi* EO.

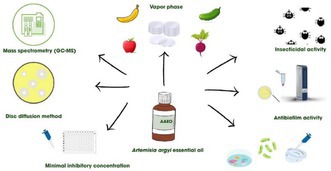

## 1 Introduction

*Artemisia argyi* H. Lév. & Vaniot, commonly known as “Aiye”, is a perennial aromatic herb belonging to the Asteraceae family. It has been used for centuries in traditional Chinese medicine (TCM) for its anti‐inflammatory, haemostatic, antimicrobial, and insect repellent properties (Liu et al. 2021). Over the past few years, interest has steadily grown in the EO derived from *A. argyi* leaves (AAEO) owing to its complex phytochemical composition and broad biological activity, especially in food preservation, phytopathogen control, and biofilm inhibition (Guan et al. 2019; Wang et al. 2006).

GC–MS analyses have revealed that AAEO is primarily composed of monoterpenes and sesquiterpenes, with major constituents such as 1,8‐cineole (eucalyptol), *α*‐thujone, *β*‐caryophyllene, borneol, artemisia ketone, and spathulenol (Guan et al. 2019; Hong et al. 2023). These compounds contribute to the oil's strong aroma and are responsible for its biological functions. The exact composition of AAEO can vary considerably depending on plant origin, time of harvest, and extraction method, which underlines the importance of chemotypic characterization prior to application in scientific and industrial contexts (Heo and Chung 2023).

In vitro studies have consistently demonstrated the antimicrobial potential of EOAA against a broad spectrum of bacterial and fungal species, including *Staphylococcus aureus* , *Escherichia coli* , *Listeria monocytogenes* , *Bacillus subtilis* , *Salmonella enterica* , *Pseudomonas aeruginosa* , and *Candida albicans* (Xiang et al. 2018). In addition, in situ applications of EOs, including *A. argyi* in some cases, such as EO vapor treatments or surface applications on minimally processed vegetables and meat, have also shown promising reductions of microbial load and extension of shelf life, confirming their potential as natural preservatives (Kamal et al. 2025).

An important property of EOs, including AAEO, is their antimicrobial activity and their potential influence on microbial adhesion and early biofilm formation, although published data on antibiofilm effects of AAEO remain very limited (Guan et al. 2019; Touati et al. 2025; Xiang et al. 2018). Studies with different EOs have shown that they can significantly reduce the biomass of biofilms formed by *S. aureus* , *Pseudomonas fluorescens* , *B. subtilis* , and *S. enterica* , even at sub‐MIC concentrations (Akbas and Kokumer 2015; Hwang et al. 2023; Xu et al. 2016). The antibiofilm effect against *S. enterica* is especially noteworthy due to this pathogen's ability to form robust biofilms on food contact surfaces and plant tissues (Palomares‐Navarro et al. 2022; Yaron and Römling 2014). The inhibition is believed to result from interference with quorum sensing and suppression of extracellular matrix production (Roy et al. 2018).

MALDI‐TOF MS has also been used in previous studies as a spectral fingerprinting tool, allowing differentiation between untreated and essential‐oil–treated bacterial samples based on changes in their mass spectral profiles (Caputo et al. 2018; Flores‐Flores et al. 2025). These changes provide indirect evidence that EO disrupts the biofilm regulatory network, making it a strong candidate for targeted action against biofilms in both food and clinical settings (Carrascosa et al. 2021).

Moreover, AAEO has demonstrated potent insecticidal activity. Fumigation and contact assays revealed toxicity against stored‐product pests such as *Tribolium castaneum* and *Plutella xylostella* (Kiran and Prakash 2015). Based on studies of other *Artemisia* species and EOs with insecticidal activity, the mode of action is often associated with neurotoxicity and inhibition of acetylcholinesterase (AChE), leading to neuromuscular dysfunction in insects (Chauhan et al. 2022; Liu et al. 2021; Srivastava et al. 2015; Tsai and Lein 2021). For AAEO specifically, the exact mode of action has not yet been elucidated and remains to be clarified in future studies.

In summary, AAEO is a multifunctional natural product with documented antimicrobial, antibiofilm, and insecticidal activity. Its broad spectrum of activity combined with its complex and adaptable chemical profile supports its application in the areas of food safety, crop protection, and integrated pest management. However, further work is needed to standardize its composition, evaluate synergistic combinations, and explore its mode of action at the molecular level (Wen et al. 2024).

Despite the increasing number of studies describing the chemical composition and basic antimicrobial activity of AAEO, several important gaps remain. Most available data are based solely on in vitro tests, while the behavior and effectiveness of AAEO directly on real food matrices have been scarcely investigated. Such conditions may substantially modify antimicrobial performance, yet they remain insufficiently explored. Furthermore, published data specifically addressing the antibiofilm activity of AAEO are very limited, and to the best of our knowledge, no study has analyzed biofilm samples treated with AAEO using MALDI‐TOF MS. In addition, data on the fumigant insecticidal activity of AAEO against major stored‐product pests, such as *Callosobruchus maculatus* , are still very limited.

This study provides several new insights into the bioactivity of AAEO. The in situ experiments on fresh food matrices reveal matrix‐dependent antimicrobial responses that are not detectable under standard laboratory conditions. MALDI‐TOF MS was used simply to compare mass spectra of biofilm samples before and after EO treatment, allowing us to observe differences in the overall spectral patterns between treated and untreated biofilms. Additionally, the fumigation tests provide new information on the susceptibility of *C. maculatus* to AAEO. Together, these findings extend the current knowledge of this EO and highlight its potential applications in food preservation and stored‐product protection.

## 2 Material and Methods

### 2.1 EO Characterization

The EO from *A. argyi* was obtained from Guilin Four Seasons Sunshine Co. Ltd. (TieShan Industrial Zones, Guilin, Guangxi, China). The oil was delivered as a pale yellow to yellowish green liquid with a characteristic herbal aroma typical of *A. argyi* (batch No.: 20231215). According to the supplier, the oil is produced by steam distillation of *A. argyi* leaves. According to the manufacturer's certificate of analysis, the oil exhibited the following physicochemical properties: relative density (20°C/20°C) ranging from 0.8940 to 0.9230, refractive index (20°C) between 1.4700 and 1.4850, and optical rotation (20°C) from −12.0° to −0.5°. The oil was stored in its original sealed container, under cool, dry, and ventilated conditions, protected from light and external odors. Its shelf life, as specified by the producer, is 36 months under appropriate storage conditions.

### 2.2 GC–MS Analysis of AAEO

AAEO was chemically investigated by gas chromatography–mass spectrometry technique. The apparatus was a Clarus 500 system (Perkin Elmer, Waltham, MA, USA) equipped with a flame ionization detector (FID) and directly coupled with a mass spectrometer. The chromatography separation of the volatiles was carried out using a Varian Factor Four VF‐5 capillary column (60 m × 0.32 mm × 1 μm). One μL of AAEO diluted in 1 mL of hexane was injected in the GC injector, set at 250°C, in split mode (1:20). The applied operative conditions were as follows: the temperature ramp started from 60°C and increased at 6°C/min to 220°C and held for 20 min. Helium served as the carrier gas at a constant flow rate of 1 mL/min. MS scans were recorded within the range 35–450 m/z using EI ionization (energy 70 eV). The ion source and the connection parts temperature were set at 180°C and 200°C, respectively. The identification of components was performed by matching their mass spectra with those reported in Wiley 2.2 and NIST 11 libraries. Further, linear retention indices (LRIs) were calculated using a homologous series of *n*‐alkanes (C₈–C_25_) injected in the same conditions and compared with available literature values. The relative amount of the identified components was expressed as a percentage of its peak area in the total ion chromatogram. No internal standards or correction factors were applied. The analyses were conducted in triplicate to ensure reproducibility.

### 2.3 Antimicrobial Activity Assessment

#### 2.3.1 Microbial Strains and Culture Conditions

The antimicrobial efficacy of AAEO was evaluated against a representative selection of bacterial and yeast strains. The bacterial set included Gram‐positive species such as *Enterococcus faecalis* (CCM 4224), *Listeria monocytogenes* (CCM 4699), and *Staphylococcus aureus* (CCM 3953), along with Gram‐negative strains including *Escherichia coli* (CCM 3954), *Salmonella enterica* subsp. *enterica* (CCM 3807), and *Yersinia enterocolitica* (CCM 7204 T). In addition, opportunistic yeast pathogens were represented by *Candida albicans* (CCM 8186), *C. glabrata* (CCM 8270), *C. krusei* (CCM 8271), *C. parapsilosis* (CCM 8260), and *C. tropicalis* (CCM 8223).

Bacterial strains were cultured in Mueller‐Hinton Broth (MHB; Oxoid, Basingstoke, UK), while yeast isolates were maintained in Sabouraud Dextrose Broth (SDB; Oxoid, Basingstoke, UK). All cultures were incubated under appropriate conditions at 37°C for bacteria and 25°C for yeasts for 24 h. Prior to antimicrobial assays, the microbial suspensions were standardized to a turbidity equivalent to 0.5 McFarland, ensuring consistency and comparability across experiments.

#### 2.3.2 Disc Diffusion Antimicrobial Assay

Preliminary evaluation of the antimicrobial activity of AAEO was carried out using the standard agar disc diffusion method. Sterile filter paper discs (6 mm in diameter) were loaded with 10 μL of the EO and placed onto Mueller–Hinton Agar (MHA, Oxoid, Basingstoke, UK) plates inoculated with bacterial cultures or Sabouraud Dextrose Agar (SDA, Oxoid, Basingstoke, UK) plates seeded with yeast suspensions, following the procedure described by Kačániová et al. (2021). All inoculated plates were incubated for 24 h at 37°C for bacterial isolates and 25°C for yeast strains. Zones of growth inhibition surrounding the discs were measured to determine antimicrobial activity. Negative control consisted of sterile discs without EO. Positive controls included cefoxitin for Gram‐positive bacteria, gentamicin for Gram‐negative bacteria, and fluconazole for yeasts (Oxoid, Basingstoke, UK).

#### 2.3.3 Determination of Minimum Inhibitory Concentrations (MIC_50_ and MIC_90_)

The minimum inhibitory concentrations (MIC_50_ and MIC_90_) of AAEO were evaluated using the broth microdilution method in sterile 96‐well microtiter plates. Due to the hydrophobic nature of AAEO, a primary stock emulsion was prepared using Tween 80, after which two‐fold serial dilutions were prepared directly in Mueller–Hinton Broth (MHB) for bacteria and Sabouraud Dextrose Broth (SDB) for yeasts. Each well was inoculated with 50 μL of microbial suspension standardized to 0.5 McFarland and 50 μL of the EO diluted in a two‐fold dilution series prepared in Mueller‐Hinton Broth for bacteria or Sabouraud Dextrose Broth for yeasts. The tested concentrations of the EO ranged from 10 to 0.00488 mg/mL, following the protocol outlined by Kačániová et al. (2023). The final concentration of Tween 80 in the wells was minimal, and a broth–Tween control was included to exclude any solvent‐related effects. Negative controls contained broth with EO but without microbial inoculum, while positive controls consisted of inoculated broth without EO. After incubation for 24 h at 37°C for bacterial strains and 25°C for yeast strains, microbial growth was measured by absorbance at 570 nm using a Glomax microplate reader (Promega, Madison, WI, USA). MIC_50_ and MIC_90_ were defined as the lowest concentrations that inhibited microbial growth by at least 50% and 90%, respectively, compared to untreated controls. All assays were performed in triplicate to guarantee reproducibility and consistency.

### 2.4 Assessment of Antimicrobial Activity of EO in Food Matrices

To mimic realistic contamination scenarios, a selection of Gram‐positive and Gram‐negative bacterial strains, as well as yeast species, was applied to fresh produce. Commercial apples, bananas, beetroots, and cucumbers were washed, cut into uniform 0.5 mm slices, and air‐dried under aseptic conditions. The prepared slices were placed in sterile 60‐mm Petri dishes and inoculated with standardized microbial suspensions (0.5 McFarland). Each slice was inoculated with 10 μL of the respective suspension, applied in three spots using a sterile pipette tip by gently touching the surface. AAEO was diluted in ethyl acetate to prepare solutions at concentrations of 500, 250, 125, and 62.5 μg/L. Ethyl acetate was selected as a volatile organic solvent that readily evaporates and enables controlled and homogeneous release of the EO into the vapor phase; EOs are not water‐soluble, and aqueous Tween 80 solutions would not volatilize and would therefore not be suitable for vapor‐phase testing. Sterile filter paper discs soaked with these solutions were affixed to the inner surfaces of the Petri dish lids, enabling vapor‐phase exposure to the inoculated samples. Control dishes contained discs treated only with ethyl acetate. All dishes were sealed with parafilm to prevent vapor leakage. Plates inoculated with bacterial strains were incubated at 37°C, whereas plates inoculated with yeasts were incubated at 25°C for 7 days. After incubation, microbial growth on the surfaces of the food matrices was visually assessed and documented by digital photography. The images were analyzed using ImageJ software (NIH, USA) to semi‐quantitatively determine the percentage of surface area covered by microbial growth, allowing evaluation of the in situ antimicrobial effect of AAEO, following the method of Kačániová et al. (2021).

### 2.5 Evaluation of Antibiofilm Activity

#### 2.5.1 Crystal Violet Assay for Biofilm Inhibition

The antibiofilm potential of AAEO was evaluated using a modified crystal violet assay following the method described by Kačániová et al. (2020). Bacterial cultures were cultivated in Mueller‐Hinton Broth (MHB) in sterile 96‐well microtiter plates. The EO was applied in two‐fold serial dilutions, resulting in final concentrations ranging from 10 to 0.0048 mg/mL. Plates were incubated statically at 37°C for 24 h. After incubation, planktonic cells were carefully removed by rinsing each well with sterile phosphate‐buffered saline (PBS). The remaining biofilms were stained with 0.1% crystal violet for 15 min, followed by thorough washing with distilled water and air drying. The biofilm‐associated dye was then solubilized in 95% ethanol, and absorbance was recorded at 570 nm. The minimum biofilm inhibitory concentration (MBIC) was identified as the lowest concentration of EO that visibly prevented biofilm formation. MBIC_50_ and MBIC_90_ values were defined as the concentrations reducing biofilm biomass by 50% and 90%, respectively, compared to untreated control wells.

#### 2.5.2 MALDI‐TOF MS Analysis of Biofilm Samples

To investigate changes in MALDI‐TOF MS spectra associated with biofilm development and exposure to AAEO, matrix‐assisted laser desorption/ionization time‐of‐flight mass spectrometry (MALDI‐TOF MS) was employed. A biofilm‐producing strain of *Salmonella enterica* was cultivated in 50 mL polypropylene tubes containing 20 mL of Mueller‐Hinton Broth (MHB), inoculated with 100 μL of a standardized bacterial suspension. Sterile coupons made of stainless steel and plastic were added to serve as biofilm growth substrates (Kačániová et al. 2023). Experimental groups were treated with 0.1% (v/v) AAEO, while untreated samples served as controls. Incubation was performed at 37°C with shaking at 170 × g for 3, 7, and 14 days. At each time point, biofilm samples were recovered from both material types, and planktonic cells from control cultures were also collected. Biofilm cells were transferred from the coupon surface onto the MALDI target and overlaid with matrix solution according to the standard protocol recommended by the instrument manufacturer (Bruker Daltonics). Mass spectral data were acquired using a Microflex LT MALDI‐TOF MS instrument (Bruker Daltonics, Germany) operated in linear positive mode within an m/z range of 2000 to 20,000. Spectral analyses were conducted using Biotyper software (Bruker), and hierarchical clustering was performed based on Euclidean distances to assess inter‐sample relationships, with replicate spectra from the same experimental condition checked for consistent grouping. No additional inferential statistical tests were applied to the clustering, which was used as an exploratory tool to visualize similarities and differences between spectra.

### 2.6 Evaluation of Insecticidal Activity

The insecticidal effect of AAEO was tested on adult specimens of *Callosobruchus maculatus* , a common storage pest. Groups of 100 beetles were placed into sterile Petri dishes lined with filter paper discs that were impregnated with 100 μL of AAEO at concentrations of 100, 50, 25, 12.5, 6.25, and 3.125%. To enhance emulsification, the EO was diluted using 0.1% polysorbate 80 (Tween 80). The dishes were sealed with parafilm to minimize vapor loss and incubated at room temperature for 24 h. Control samples included filter papers treated solely with 0.1% polysorbate. After the exposure period, the numbers of live and dead beetles in each group were recorded. All tests were performed in triplicate. Mortality rates were calculated to evaluate the dose‐dependent insecticidal effect of the EO under closed system conditions.

### 2.7 Statistical Analysis

Data processing and graphical presentations were carried out using JMP Student Edition version 18.0 (SAS Institute, Cary, NC, USA). All analyses were performed on three independent replicates (*n* = 3) for each sample. Results are reported as mean values ± standard deviation (SD). Prior to statistical testing, data were checked for normal distribution (Shapiro–Wilk test) and homogeneity of variances (Levene's test). When assumptions were met, differences among groups were evaluated using one‐way ANOVA, followed by Tukey's HSD post hoc test. Statistical significance was set at *p* ≤ 0.05.

## 3 Results

### 3.1 Chemical Composition of *Artemisia argyi* Essential Oil

The GC–MS analyses identified and semi‐quantified 59 components in AAEO listed in Table 1. The chemical profile was characterized by a prevalence of monoterpenes (46.1%) respect to sesquiterpenes (33.7%). 1,8‐Cineole was the most abundant monoterpene (20.5%) while among the sesquiterpenes *β*‐caryophyllene (16.5%) and longifolene (13.8%) prevailed.

**TABLE 1:** Chemical composition of *Artemisia argyi* EO (percentage mean values).

N.	Components	LRI^calc^	LRI^lit^	%
1	Santolina triene	900	901	tr
2	*α*‐Pinene	938	941	3.0
3	Camphene	940	943	0.5
4	Dehydrosabinene	951	955	0.3
5	*β*‐Thujene	960	968	0.2
6	Pseudolimonene	989	993	0.4
7	2,3‐Dehydro‐1,8‐cineole	991	994	0.2
8	*β*‐pinene	970	975	2.4
9	(*E*)‐3,6‐Heptadien‐2‐ol,2,5,5‐trimethyl	1002	1000	0.4
10	*α*‐Phellandrene	1006	1005	0.4
11	*α*‐Terpinene	1021	1019	0.7
12	*p*‐Cymene	1024	1022	2.0
13	1,8‐Cineole	1035	1033	20.5
14	Artemisia ketone	1040	1041	0.3
15	4‐Thujanol	1048	1051	0.5
16	*γ*‐Terpinene	1063	1062	1.4
17	Artemisia alcohol	1071	1068	1.2
18	Terpinolene	1072	1076	0.3
19	Linalool	1088	1092	2.4
20	*trans*‐Chrysanthenol	1110	1114	0.1
21	*β*‐Thujone	1115	1116	0.8
22	Chrysanthenone	1118	1120	0.2
23	*α*‐Thujone	1122	1124	3.0
24	*cis‐β*‐Terpineol	1124	1126	0.5
25	*p*‐Menth‐2‐en‐1‐ol	1130	1127	0.3
26	*trans*‐Sabinol	1135	1140	0.2
27	*cis*‐*p*‐Menth‐2‐en‐1‐ol	1140	1145	0.3
28	D‐Camphor	1152	1151	4.1
29	*β*‐Artemisia‐acetate	1154	1152	0.7
30	*p*‐Menthan‐8‐ol	1160	1160	0.4
31	*cis*‐Chrysanthenol	1164	1161	1.6
32	Endo‐borneol	1169	1166	2.9
33	Terpinen‐4‐ol	1185	1182	5.6
34	*α*‐Terpineol	1189	1193	3.4
35	2‐Pinen‐4‐one	1192	1196	0.2
36	*cis*‐Piperitol	1212	1215	0.2
37	*trans*‐Carveol	1216	1218	0.8
38	Carvone	1241	1243	0.1
39	Piperitone	1258	1254	0.1
40	*cis*‐Chrysanthenyl acetate	1271	1266	0.1
41	Perillal	1275	1271	0.2
42	Bornyl acetate	1282	1280	0.3
43	Isobornyl acetate	1288	1284	0.1
44	Eugenol	1352	1350	0.2
45	Cyclosativene	1371	1368	0.2
46	Ylangene	1378	1376	0.1
47	*α*‐Copaene	1380	1379	0.2
48	Longicyclene	1389	1392	1.6
49	β‐Elemene	1395	1393	0.2
50	Sativene	1397	1396	0.5
51	Isocaryophyllene	1410	1407	0.2
52	Longifolene	1422	1418	13.8
53	*β*‐Caryophyllene	1434	1435	16.5
54	(*E)‐β*‐farnesene	1452	1447	0.4
55	Humulene	1476	1473	0.1
56	Germacrene D	1505	1500	0.5
57	*β*‐Eudesmene	1511	1509	0.4
58	δ‐Amorphene	1542	1538	0.4
59	Caryophyllene oxide	1591	1585	1.1
	Total			99.7
	Monoterpenes Hydrocarbons			46.1
	Sesquiterpenes Hydrocarbons			33.7
	Terpenoids			16.1
	Others			3.8

*Note:* The components are reported according to their elution order on apolar column; LRI^calc^, Linear Retention Indices measured on apolar column; LRI^lit^, Linear Retention indices from literature (Nist WebBook), tr, percentage mean values less than 0.1%.

### 3.2 Antimicrobial Activity In Vitro

The antimicrobial activity of AAEO was assessed using the disc diffusion method against various microorganisms, with results summarized in the Table 2. Among Gram‐negative bacteria, AAEO produced inhibition zones ranging from 8.67 mm for *Y. enterocolitica* to 11.67 mm for *S. enterica* , while *E. coli* showed an inhibition zone of 10.67 mm. For Gram‐positive bacteria, the EO demonstrated stronger activity, with the largest inhibition zones observed for *S. aureus* (16.33 mm), followed by *L. monocytogenes* (15.33 mm) and *E. faecalis* (13.67 mm). Against yeasts of the *Candida* genus, inhibition zones ranged from 7.67 mm for *C. glabrata* to 10.33 mm for *C. tropicalis* , with other *Candida* species showing intermediate values. The biofilm‐forming strain of *S. enterica* was inhibited with an average zone of 12.33 mm. In comparison, the antibiotic controls (ATB) showed significantly larger inhibition zones across all tested microorganisms, ranging from 27.33 mm ( *C. tropicalis* ) to 32.33 mm ( *S. enterica* ). All values represent means ± standard deviations of three replicates. Statistically significant differences between microorganisms were indicated (*p* ≤ 0.05) as shown by differing superscript letters in the Table 2. These results indicate that AAEO exhibits moderate antimicrobial activity, with greater efficacy against Gram‐positive bacteria than against Gram‐negative bacteria and yeasts. The observed inhibition of biofilm‐forming *S. enterica* suggests a potential role of AAEO as an adjunct antimicrobial agent against microbial pathogens.

**TABLE 2:** Antimicrobial activity is assessed by the disc diffusion method.

Microorganism	Inhibition zone of AAEO (mm)	Inhibition zone of ATB (mm)
*G‐negative bacteria*		
*Escherichia coli* CCM 3954	10.67 ± 0.58^def^	29.67 ± 0.59^bcd^
*Salmonella enterica* CCM 3807	11.67 ± 0.58^de^	32.33 ± 0.58^a^
*Yersinia enterocolitica* CCM 7204T	8.67 ± 0.58^ghi^	30.33 ± 0.58^bc^
*G‐positive bacteria*		
*Enterococcus faecalis* CCM 4224	13.67 ± 0.58^bc^	31.33 ± 0.59^ab^
*Listeria monocytogenes* CCM 4699	15.33 ± 0.58^ab^	30.67 ± 0.58^abc^
*Staphylococcus aureus* CCM 4423	16.33 ± 0.58^a^	31.33 ± 0.58^ab^
*Yeast*		
*Candida albicans* CCM 8186	8.33 ± 0.58^hi^	29.67 ± 0.59^bcd^
*Candida glabrata* CCM 8270	7.67 ± 0.58^i^	29.33 ± 0.58^cd^
*Candida krusei* CCM 8271	9.33 ± 0.58^fghi^	28.33 ± 0.58^de^
*Candida parapsilosis* CCM 8260	9.67 ± 0.58^fgh^	30.33 ± 0.59^bc^
*Candida tropicalis* CCM 8223	10.33 ± 0.58^efg^	27.33 ± 0.57^e^
*BFB*		
Biofilm forming *S. enterica*	12.33 ± 0.58^cd^	29.33 ± 0.58^cd^

*Note:* Data represents the mean (± standard deviation) (*n* = 3). Different letters in each column indicate significant differences (*p* ≤ 0.05).

Abbreviations: AAEO, *Artemisia argyi* essential oil; ATB, Antibiotics.

MIC_50_ and MIC_90_ of AAEO were determined against various microorganisms. For G^−^ bacteria, MIC_50_ ranged from 2.46 mg/mL for *Y. enterocolitica* to 5.41 mg/mL for *E. coli* . *S. enterica* showed an MIC_50_ of 2.79 mg/mL. Among G^+^ bacteria, the lowest MIC_50_ was observed for *E. faecalis* (1.16 mg/mL), while *L. monocytogenes* required 5.63 mg/mL. *S. aureus* had an MIC_50_ of 3.31 mg/mL. For yeasts, MIC_50_ was lowest for *C. albicans* (0.30 mg/mL) and highest for *C. parapsilosis* (9.49 mg/mL), with other species showing intermediate values. This wide range reflects species‐specific differences in susceptibility among the tested *Candida* strains; non‐*albicans* species such as *C. krusei* and *C. parapsilosis* are generally more tolerant to antimicrobial agents than *C. albicans* , and all strains were tested under identical experimental conditions, so the variability is most likely due to intrinsic biological differences rather than methodological error.

### 3.3 In Situ Antimicrobial Activity

**FIGURE 1:**
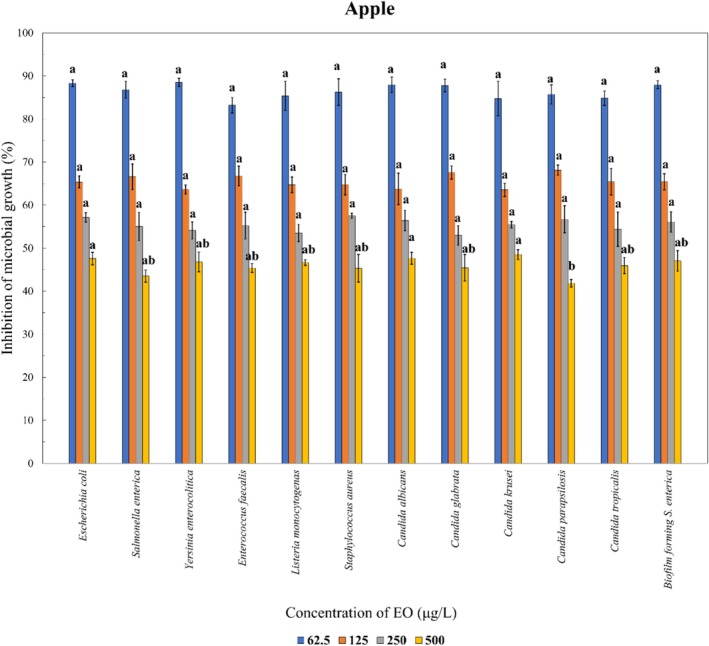
In situ analysis of the inhibition of microbial growth (%) of the vapor phase of AAEO in apple. Data are the mean (bars indicate ± standard deviation) (*n* = 3). Different letters for each EO concentration refer to significant differences (*p ≤* 0.05).

On apple, the highest inhibition of microbial growth was observed at the lowest tested concentration of 62.5 μg/L of AAEO (Figure 1). The strongest inhibition was seen against *Y. enterocolitica* (88.51%), followed by *E. coli* (88.34%) and biofilm‐forming *S. enterica* (87.95%). At 125 μg/L EO, the inhibition slightly decreased: *E. coli* showed 65.37%, *Y. enterocolitica* 63.63%, and biofilm‐forming *S. enterica* 65.41%. Further increase of EO concentration to 250 μg/L led to a continued drop in inhibition *E. coli* at 57.14%, *Y. enterocolitica* 54.10%, and biofilm‐forming *S. enterica* 56.08%. At the highest tested EO concentration of 500 μg/L, inhibition was the lowest: *E. coli* 47.60%, *Y. enterocolitica* 46.80%, and biofilm‐forming *S. enterica* 47.03%. This decreasing trend with increasing EO concentration was also observed for other microorganisms, including G‐positive bacteria ( *E. faecalis* , *L. monocytogenes* , *S. aureus* ) and yeasts ( *C. albicans* , *C. glabrata* , *C. krusei* , *C. parapsilosis* , *C. tropicalis* ). The lowest inhibition at 500 μg/L EO was recorded for *C. parapsilosis* with 41.84%, while the highest inhibition at this concentration remained with biofilm‐forming *S. enterica* at 47.03%. Overall, AAEO inhibited microbial growth on apple best at the lowest tested concentration of 62.5 μg/L, with effectiveness decreasing as concentration increased.

**FIGURE 2:**
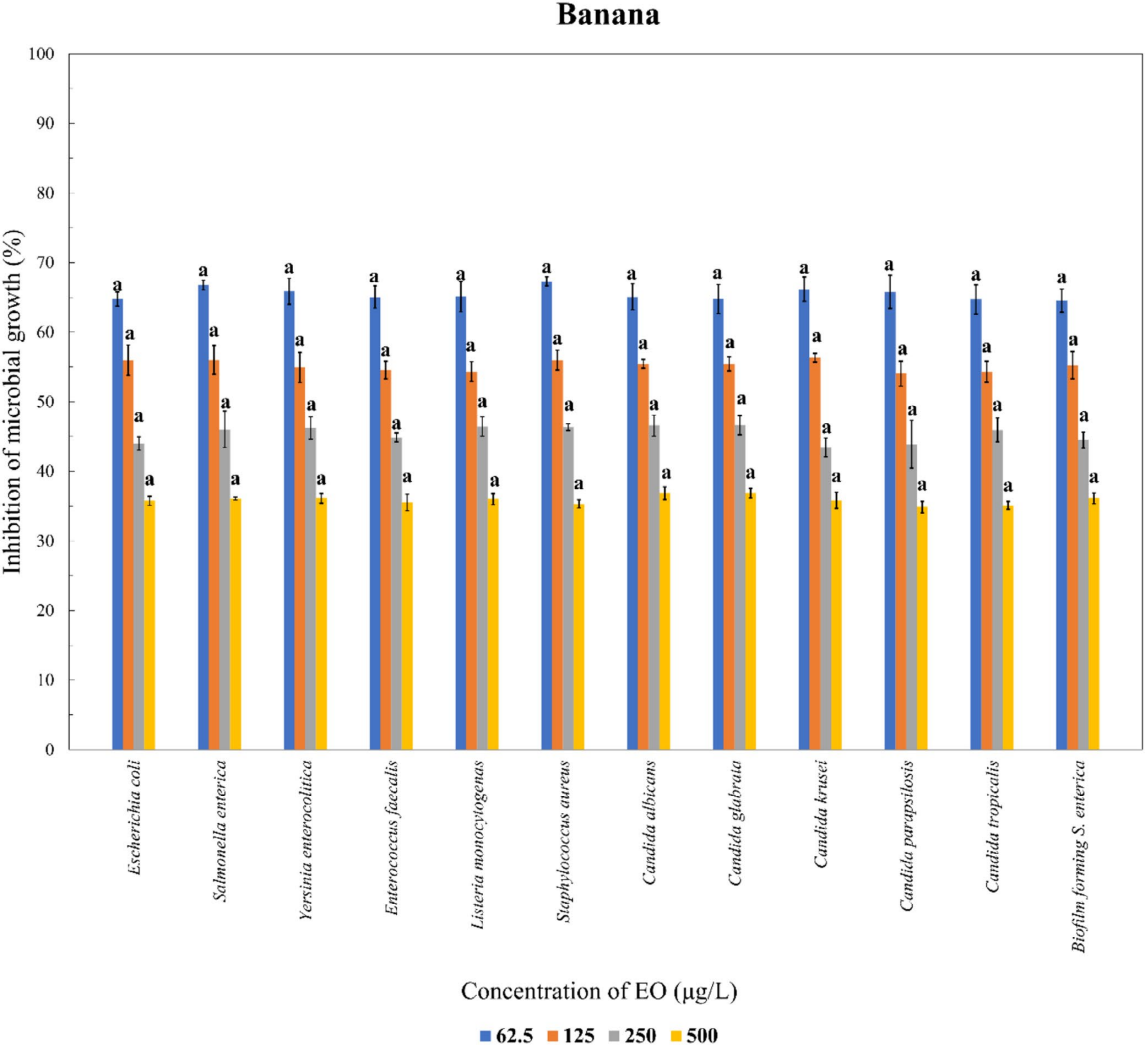
In situ analysis of the inhibition of microbial growth (%) of the vapor phase of AAEO in banana. Data are the mean (bars indicate ± standard deviation) (*n* = 3). Different letters for each EO concentration refer to significant differences (*p ≤* 0.05).

On banana, the highest inhibition of microbial growth was seen at the lowest tested AAEO concentration of 62.5 μg/L (Figure 2). Among G^−^ bacteria, inhibition was *E. coli* 65.41%, *S. enterica* 67.12%, and *Y. enterocolitica* 66.69%. For G^+^ bacteria, inhibition at 62.5 μg/L EO was *E. faecalis* 57.67%, *L. monocytogenes* 55.20%, and *S. aureus* 56.93%. Yeasts from the *Candida* genus showed inhibition between 54.49% and 57.46%, with *C. albicans* at 55.94% and *C. tropicalis* at 55.48%. Biofilm‐forming *S. enterica* was inhibited by 66.17% at 62.5 μg/L EO. As EO concentration increased to 125 μg/L, inhibition dropped slightly to *E. coli* 54.79%, *S. enterica* 55.64%, *Y. enterocolitica* 54.77%, *E. faecalis* 53.19%, *L. monocytogenes* 49.60%, *S. aureus* 53.75%, *C. albicans* 51.37%, *C. tropicalis* 52.52%, and biofilm‐forming *S. enterica* 56.42%. At 250 μg/L EO, inhibition decreased further to *E. coli* 49.88%, *S. enterica* 50.81%, *Y. enterocolitica* 48.45%, *E. faecalis* 45.53%, *L. monocytogenes* 43.76%, *S. aureus* 46.41%, *C. albicans* 43.55%, *C. tropicalis* 43.67%, and biofilm‐forming *S. enterica* 52.21%. At the highest EO concentration of 500 μg/L, inhibition was lowest for *E. coli* 35.78%, *S. enterica* 36.42%, *Y. enterocolitica* 36.19%, *E. faecalis* 34.92%, *L. monocytogenes* 32.99%, *S. aureus* 35.41%, *C. albicans* 34.92%, *C. tropicalis* 34.76%, and biofilm‐forming *S. enterica* 36.25%.

**FIGURE 3:**
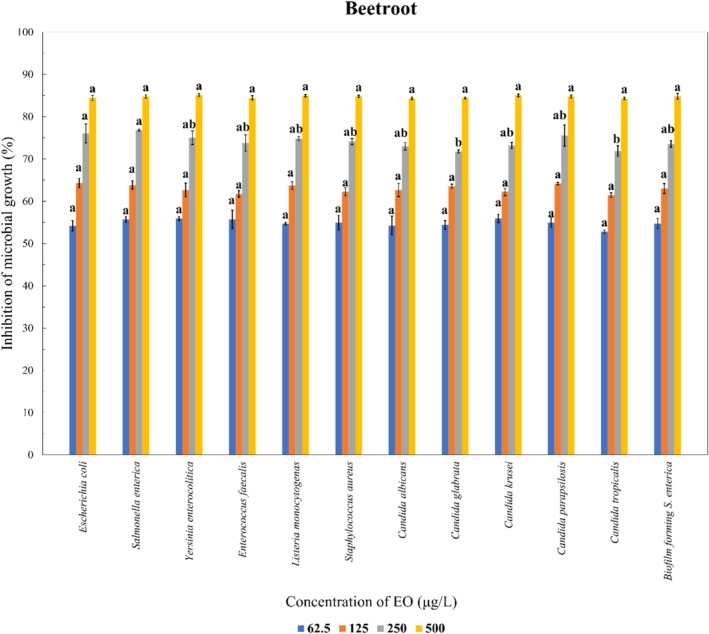
In situ analysis of the inhibition of microbial growth (%) of the vapor phase of AAEO in beetroot. Data are the mean (bars indicate ± standard deviation) (*n* = 3). Different letters for each EO concentration refer to significant differences (*p ≤* 0.05).

On beetroot (Figure 3), microbial growth inhibition at an EO concentration of 62.5 μg/L ranged approximately from 54.17% ( *E. coli* ) to 55.92% ( *C. krusei* ). At 125 μg/L EO, inhibition ranged from 61.73% ( *E. faecalis* ) to 64.31% ( *E. coli* ). Increasing the EO concentration to 250 μg/L led to a further increase in inhibition, ranging between 71.76% ( *C. glabrata* ) and 76.04% ( *E. coli* ). The highest tested EO concentration of 500 μg/L reached microbial growth inhibition between 84.37% ( *C. albicans* ) and 85.09% ( *Y. enterocolitica* ). Among G^−^ bacteria, the highest inhibition at 500 μg/L EO was observed in *Y. enterocolitica* (85.09%), while among G^+^ bacteria, the strongest growth suppression at the same EO concentration was in *E. faecalis* (84.49%) and *L. monocytogenes* (84.99%). Biofilm‐forming *S. enterica* reached 84.83% inhibition at 500 μg/L EO. Overall data show that microbial growth inhibition on beetroot significantly increases with rising EO concentration, with the highest efficacy recorded at 500 μg/L EO.

**FIGURE 4:**
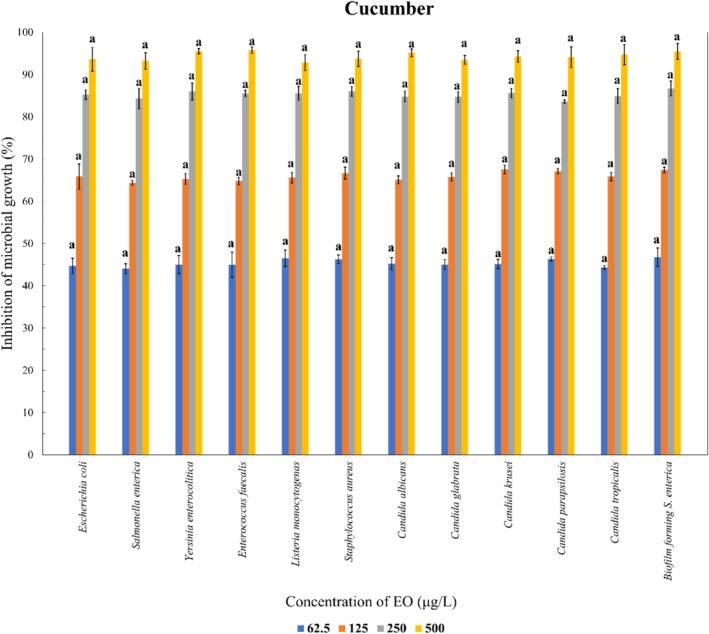
In situ analysis of the inhibition of microbial growth (%) of the vapor phase of AAEO in cucumber. Data are the mean (bars indicate ± standard deviation) (*n* = 3). Different letters for each EO concentration refer to significant differences (*p ≤* 0.05).

On cucumber (Figure 4), microbial growth inhibition at the lowest EO concentration of 62.5 μg/L ranged from 46.37% ( *E. coli* ) to 49.18% ( *C. tropicalis* ). At 125 μg/L EO, inhibition ranged from 53.65% ( *E. faecalis* ) to 58.82% ( *C. albicans* ). At 250 μg/L EO, inhibition increased, ranging from 62.22% ( *C. glabrata* ) to 66.19% ( *E. coli* ). The highest tested EO concentration of 500 μg/L reached microbial growth inhibition between 67.91% ( *E. faecalis* ) and 70.96% ( *E. coli* ). Among G^−^ bacteria, the highest inhibition at 500 μg/L EO was observed in *E. coli* (70.96%), while among G^+^ bacteria, *L. monocytogenes* (70.72%) and *S. aureus* (69.46%) showed the strongest growth suppression. Yeasts of the *Candida* genus exhibited inhibition ranging from 67.15% ( *C. krusei* ) to 69.69% ( *C. tropicalis* ) at 500 μg/L EO. Biofilm‐forming *S. enterica* was inhibited by 68.61% at 500 μg/L EO.

### 3.4 Antibiofilm Activity of AAEO

**FIGURE 5:**
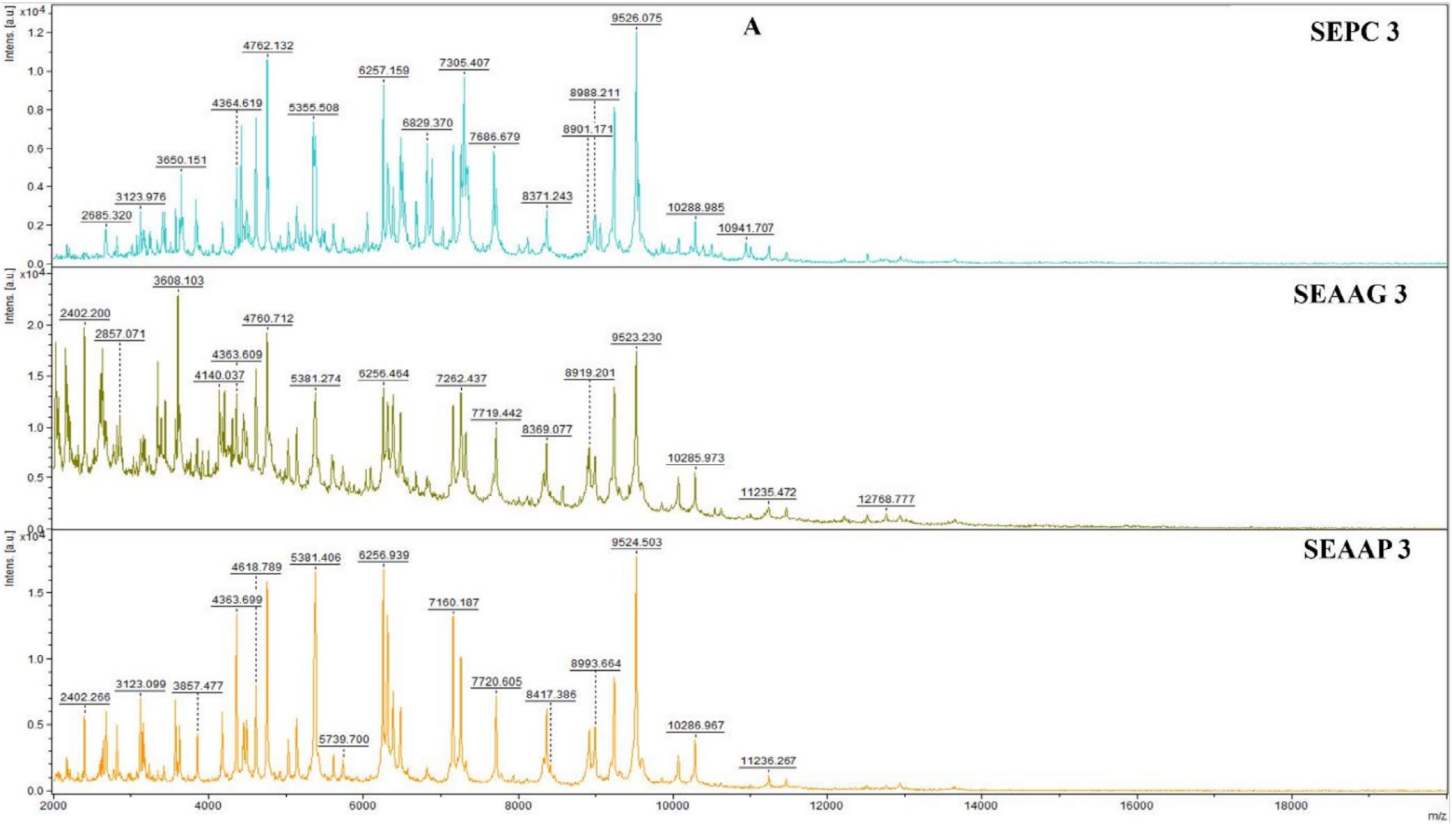
MALDI‐TOF MS spectra of *S. enterica* biofilms formed on plastic (SEAAP 3), glass (SEAAG 3), and planktonic cells (SEPC 3) after 3 days of incubation.

Biofilm‐forming *S. enterica* exhibited an MBIC_50_ of 1.51 mg/mL. MBIC_90_ values were slightly higher but followed a similar trend as MIC_50_ (Table 3). MALDI‐TOF MS spectra of the microorganism *S. enterica* after 3 days of incubation were analyzed under three different conditions (Figure 5): biofilm formed on a plastic surface (P), biofilm on a glass surface (G), and planktonic cells (PC). The spectra of all three samples showed a high degree of similarity, with major peaks matching in m/z values and intensities. No additional or missing peaks were detected, indicating that within the first 3 days, only minimal spectral differences developed between surfaces or between biofilm and planktonic forms. These results suggest that after 3 days of growth, *S. enterica* does not exhibit significant changes in protein profile between biofilms on plastic and glass surfaces or compared to planktonic cells when analyzed by MALDI‐TOF MS. The stability of spectra across different conditions indicates that, within this time frame, the microorganism's protein profiles remain relatively consistent without notable variation depending on the type of surface or growth form.

**TABLE 3:** Minimal inhibition concentration (MIC_50_ and MIC_90_) of AAEO in mg/mL.

Microorganism	MIC_50_	MIC_90_
*G‐negative bacteria*		
*Escherichia coli* CCM 3954	5.409 ± 0.401^c^	5.614 ± 0.408^c^
*Salmonella enterica* CCM 3807	2.792 ± 0.100^de^	2.944 ± 0.043^de^
*Yersinia enterocolitica* CCM 7204T	2.460 ± 0.106^e^	2.638 ± 0.167^e^
*G‐positive bacteria*		
*Enterococcus faecalis* CCM 4224	1.159 ± 0.085^f^	1.310 ± 0.115^f^
*Listeria monocytogenes* CCM 4699	5.632 ± 0.424^c^	5.908 ± 0.365^c^
*Staphylococcus aureus* CCM 4423	3.309 ± 0.166^d^	3.498 ± 0.161^d^
*Yeast*		
*Candida albicans* CCM 8186	0.296 ± 0.050^g^	0.379 ± 0.040^g^
*Candida glabrata* CCM 8270	1.459 ± 0.074^f^	1.705 ± 0.129^f^
*Candida krusei* CCM 8271	7.706 ± 0.241^b^	7.856 ± 0.187^b^
*Candida parapsilosis* CCM 8260	9.485 ± 0.234^a^	9.592 ± 0.257^a^
*Candida tropicalis* CCM 8223	2.645 ± 0.313^de^	2.748 ± 0.316^e^
*BFB*	MBIC_50_	MBIC_90_
Biofilm forming *S. enterica*	1.509 ± 0.210^f^	1.639 ± 0.238^f^

*Note:* Data represents the mean (± standard deviation) (*n* = 3). Different letters in each column indicate significant differences (*p* ≤ 0.05).

**FIGURE 6:**
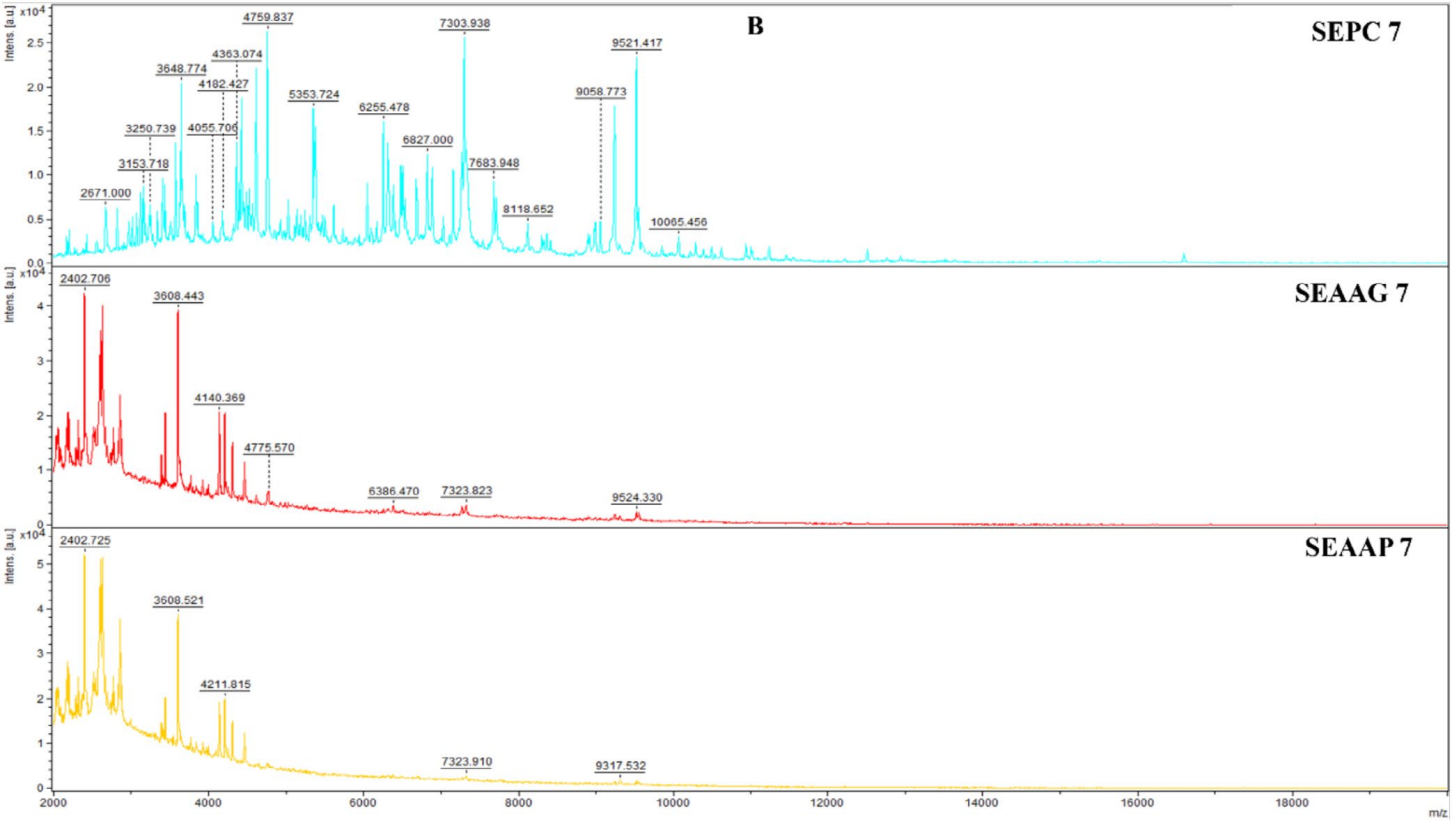
MALDI‐TOF MS spectra of *S. enterica* biofilms formed on plastic (SEAAP 7), glass (SEAAG 7), and planktonic cells (SEPC 7) after 7 days of incubation.

The MALDI‐TOF MS spectra of *Salmonella enterica* after 7 days of incubation were analyzed for two test conditions (Figure 6): biofilm on a glass surface (SEAAG 7) and biofilm on a plastic surface (SEAAP 7), with planktonic cells (SEPC 7) serving as the control. The planktonic cells spectrum shows high peak intensities in the range of approximately 2400–10,000 m/z. The biofilm on glass displays similar peaks but with significantly reduced intensities, especially in the higher m/z range. The biofilm on plastic exhibits the lowest peak intensities and the simplest spectral profile among all three groups. These results suggest that the molecular composition of the *S. enterica* biofilm varies depending on the surface type. The biofilm on plastic shows more pronounced changes compared to the biofilm on glass, while planktonic cells represent the reference state with the highest spectral complexity.

**FIGURE 7:**
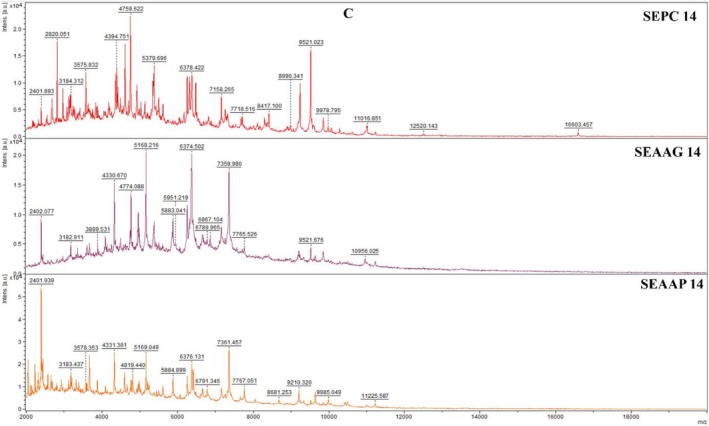
MALDI‐TOF MS spectra of *S. enterica* biofilms formed on plastic (SEAAP 14), glass (SEAAG 14), and planktonic cells (SEPC 14) after 14 days of incubation.

MALDI‐TOF MS spectra of *S. enterica* after 14 days of incubation were analyzed for planktonic cells (SEPC 14) and biofilms formed on glass (SEAAG 14) and plastic surfaces (SEAAP 14) (Figure 7). The spectrum of planktonic cells shows the highest peak intensity and complexity, reflecting the standard state of the microorganism without surface influence. The biofilm on glass exhibits similar spectral features but with a slight decrease in peak intensities, indicating some microbial adaptation during growth on this surface. The biofilm on plastic shows the lowest intensity and the simplest spectral profile, suggesting more pronounced changes in the molecular state of the microorganism compared to the other conditions. These results indicate that after 14 days of growth, *S. enterica* undergoes adaptive molecular changes depending on the biofilm surface type. Planktonic cells serve as a reference standard, while the changes observed in biofilms reflect different biological responses of the microorganism to varying environments.

**FIGURE 8:**
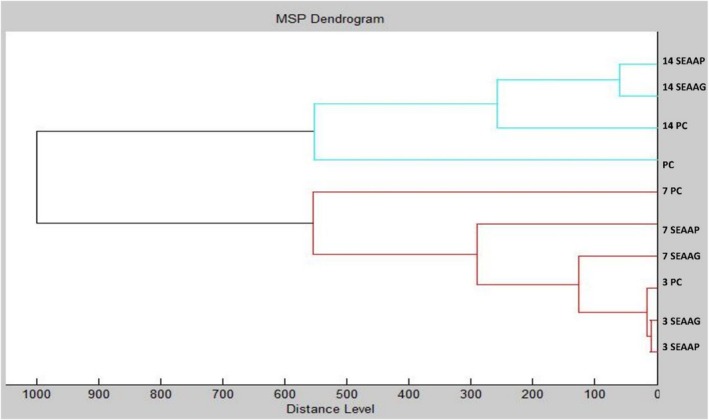
MSP dendrogram illustrating the clustering of *S. enterica* samples based on MALDI‐TOF MS spectral profiles after 3, 7, and 14 days of incubation in planktonic cells (PC) and biofilms formed on plastic (SEAAP) and glass (SEAAG) surfaces.

The biofilm of *Salmonella enterica* changed primarily depending on the incubation duration and the surface on which it developed (Figure 8). After 3 days of incubation, the MALDI‐TOF MS spectra of biofilms formed on plastic and glass surfaces were very similar to the spectra of planktonic cells, indicating that at this early stage, there were no significant molecular structural changes in the biofilm depending on the surface. This suggests relative homogeneity of the biofilms in the initial phase of development. Over time, after 7 days, differences began to emerge between biofilms on different surfaces. The biofilm on the plastic surface was markedly different from the biofilm on glass and from planktonic cells, as evidenced by changes in peak intensities in the MALDI‐TOF MS spectra. These changes may be related to the adaptation of microorganisms to the specific physicochemical properties of the surface, which influence the structure and composition of the biofilm. After 14 days, these differences became even more pronounced, with the biofilm on the plastic surface showing the simplest and weakest spectral profile, which may be a result of long‐term adaptation and changes in the metabolic activities of the microorganisms within the biofilm.

In this context, the effect of AAEO on *S. enterica* biofilm was also investigated. AAEO exhibits significant antibacterial activity, which can affect the formation and composition of the biofilm. The action of AAEO leads to a reduction in the intensity of certain peaks in the spectrum, indicating the suppression of selected molecular components of the biofilm. This suggests that AAEO can effectively disrupt the biofilm, which has practical implications for controlling bacterial infections and contamination on various surfaces.

The dendrogram based on MALDI‐TOF MS spectra of *S. enterica* at three different time points (3, 7, and 14 days) and under three conditions (planktonic cells, biofilm on glass surface, and biofilm on plastic surface) revealed clear differences and temporal changes in spectral profiles. Planktonic cells from all time points clustered very closely, indicating high stability of their protein spectra throughout the incubation period. In contrast, biofilms on glass and plastic surfaces formed two separate branches in the dendrogram, clearly reflecting the influence of surface type on the protein composition of the biofilm. Within the biofilm groups, temporal dynamics were also evident: spectra from days 3 and 7 were closer to each other, while those from day 14 were more distant, indicating progressive changes in the biofilm over the incubation period. The biofilm formed on the plastic surface differed from that on glass, suggesting surface‐specific microbial adaptations. These findings correspond with the MALDI‐TOF MS analysis results, where similar trends in peak intensity and complexity changes were observed depending on time and surface. Overall, the dendrogram confirms that the MALDI‐TOF MS spectral profiles of *S. enterica* are influenced not only by the type of surface on which the biofilm grows but also by the length of incubation, while planktonic cells remain spectral profiles stable throughout the monitored period.

### 3.5 Insecticidal Activity of AAEO

The insecticidal activity of AAEO against *C. maculatus* was concentration dependent (Table 4). The highest concentration (100%) caused almost complete mortality (99.00% ± 1.00%), which was significantly higher than all other treatments. At 50% concentration, mortality decreased to 88.00% ± 2.13%, followed by 77.00% ± 3.02% at 25%. A further decrease was observed at 12.5%, where mortality dropped to 31.00% ± 1.54%. Low concentrations (6.25% and 3.125%) showed only minimal or no effect, with mortality values of 7.00% ± 3.33% and 0.50% ± 0.50%, respectively, which were statistically comparable to the control. These results demonstrate that AAEO exhibits strong fumigant insecticidal activity at concentrations of 50% and above, whereas its effectiveness is limited at lower concentrations.

**TABLE 4:** Insecticidal activity of AAEO against *Callosobruchus maculatus* .

Concentration (%)	Insecticidal activity (%)
100	99.00 ± 1.00^a^
50	88.00 ± 2.13^b^
25	77.00 ± 3.02^c^
12.5	31.00 ± 1.54^d^
6.25	7.00 ± 3.33^e^
3.125	0.50 ± 0.50^f^
Control group	0.00 ± 0.00^f^

*Note:* Data are the mean (±SD) (*n* = 3). Different letters in the column indicate significant differences (*p* ≤ 0.05).

## 4 Discussion

The chemical analysis of the AAEO revealed a complex composition with a high proportion of oxidized compounds. Major constituents included 1,8‐cineole (20.5%), *β*‐caryophyllene (16.5%), and longifolene (13.8%), which together comprised nearly half of the total composition. Other significant components were terpinen‐4‐ol (5.6%), D‐camphor (4.1%), *α*‐pinene (3.0%), and *α*‐thujone (3.0%). These findings are consistent with literature data reporting 1,8‐cineole as the dominant compound in AAEO, typically ranging from 10% to 30% (Guan et al. 2019; Huang et al. 2012; Li et al. 2023; Ozek et al. 2014; Shan and Zhou 2021; Wenqiang et al. 2006; Xiang et al. 2018; Zhang et al. 2014). The high proportion of oxidized compounds is also characteristic of hydrolates derived from these oils, which predominantly contain oxidized derivatives with low hydrocarbon content, an important factor for their biological activity (Aćimović et al. 2022; Chizzola et al. 2021; Tomi et al. 2016). *β*‐Caryophyllene and longifolene (16.5% and 13.8%, respectively) align with reported values in the literature, where these sesquiterpenes are associated with the anti‐inflammatory and antimicrobial properties of EOs (Luo et al. 2022). The low levels of *α*‐ and *β*‐thujone (3.0% and 0.8%) are favorable from a safety perspective, as higher concentrations of these compounds may exert toxic effects (Chen et al. 2017; Luo et al. 2022; Xiang et al. 2018). The pharmacological significance of 1,8‐cineole includes a broad spectrum of activities such as anti‐inflammatory, antimicrobial, and antineoplastic effects demonstrated in both in vitro and in vivo studies (Cai et al. 2021). The combination of 1,8‐cineole with camphor, borneol, and other terpenoids contributes to the overall bioactivity of AAEO (Chen et al. 2017; Ehrnhöfer‐Ressler et al. 2013). Based on the chemical profile, the analyzed EO exhibits potential for applications in antimicrobial protection and anti‐inflammatory therapy, with its low thujone content enhancing the safety profile for possible uses.

Our results confirm that the AAEO exhibits moderate antimicrobial activity, with stronger efficacy against Gram‐positive bacteria compared to Gram‐negative bacteria and yeasts. This trend corresponds with the literature, where Gram‐positive pathogens are generally more sensitive to plant EOs (Guan et al. 2019). The most sensitive Gram‐positive species in our experiment was *S. aureus* , with an inhibition zone exceeding 16 mm, which aligns with previous studies reporting low MIC values for this species, often ranging between 0.3 and 0.6 mg/mL (Li et al. 2024). The Gram‐negative *E. coli* was less sensitive, yet inhibition zones above 10 mm indicate that AAEO also has potential activity against it. The biofilm‐forming strain of *S. enterica* was inhibited with an average zone of 12.33 mm and exhibited an MBIC_50_ of 1.51 mg/mL, suggesting that AAEO may act against biofilms, which are known for their increased resistance to antimicrobial agents. These findings are crucial for the potential application of AAEO in food preservation and healthcare as an adjunct antimicrobial agent. The literature also emphasizes the importance of active compounds with high diffusibility in agar media, which significantly affects the antimicrobial efficacy of plant EOs (Danh et al. 2013). Our results demonstrate that the combination of inhibition zones and MIC values indicates that AAEO is particularly effective against Gram‐positive bacteria, supporting further research and potential applications. Overall, these results confirm that AAEO is a promising natural antimicrobial agent suitable for combating a wide range of pathogens, including biofilm‐forming strains. Similar results have been reported by (Diao et al. 2014). These results revealed that the active compounds from EO might bind to the cell surface and then penetrate into the target sites, which could destroy the structure of cell walls.

While in vitro testing provides essential baseline data on the antimicrobial potential of AAEO, it is necessary to evaluate its efficacy under real conditions that better reflect practical applications (Burt 2004; Hyldgaard et al. 2012). Thus, in situ antimicrobial activity was assessed on four different food matrices: apple, banana, beetroot, and cucumber to simulate realistic conditions of storage and distribution. The results revealed a significant dependence of antimicrobial efficacy on both the type of food matrix and the EO concentration (Khan et al. 2023).

A particularly notable and unexpected finding was observed on apple and banana, where the highest inhibition of microbial growth was recorded at the lowest tested concentration of 62.5 μg/L AAEO. Increasing the concentration of EO led to a marked decrease in antimicrobial efficacy. One possible explanation is a matrix‐dependent phytotoxic effect at higher concentrations, which may damage the surface of plant tissues and release nutrients or cellular fluids that support microbial growth. This phenomenon has been described for several EOs on fresh produce, where tissue injury at higher doses can counteract their antimicrobial action (Ali et al. 2025). Another contributing factor may be physicochemical saturation effects. At higher EO concentrations, rapid condensation of volatile constituents on the food surface may reduce the amount of active vapor‐phase compounds available to exert antimicrobial action, as suggested in previous studies on vapor‐phase EOs (Sánchez‐González et al. 2011). These combined mechanisms, phytotoxic surface responses at high doses and reduced vapor‐phase diffusion due to condensation, may explain why lower concentrations produced stronger inhibitory effects in apple and banana. Such behavior cannot be captured in standard in vitro assays, underscoring the relevance of in situ testing for understanding real‐matrix interactions.

Conversely, on beetroot and cucumber, antimicrobial activity increased proportionally with EO concentration, consistent with conventional models of EO action observed in vitro (Burt 2004). These contrasting patterns emphasize the influence of matrix‐specific physical and chemical properties such as pH, moisture content, and surface characteristics on EO release and antimicrobial effectiveness (Pedreira et al. 2024).

The ability of AAEO to significantly inhibit growth of Gram‐negative bacteria ( *E. coli* , *Y. enterocolitica* , *S. enterica* ), including biofilm‐forming strains, across all tested matrices is especially noteworthy. Gram‐negative pathogens and biofilms are well known for their resistance to many antimicrobial agents, posing a significant challenge in food safety (Donlan 2002). The in situ confirmation of AAEO efficacy against these organisms strengthens its potential as a natural preservative in food industry applications.

Comparing the in vitro and in situ data, it is evident that although AAEO demonstrates promising antimicrobial properties in controlled laboratory conditions, real‐world efficacy is modulated by matrix interactions and volatile compound behavior in complex food environments. This highlights the necessity for in situ testing as a critical step bridging basic research and application, providing insights into optimal concentrations and formulations for different food products.

In conclusion, our integrated approach validates AAEO as a multifunctional natural antimicrobial agent with applicability in food preservation, particularly when matrix‐specific factors are considered. Future work should focus on elucidating the mechanisms governing EO release and interaction within food matrices and optimizing application strategies to maximize efficacy while maintaining food quality.

The biofilm‐forming strain of *S. enterica* exhibited an MBIC_50_ value of 1.51 mg/mL, with MBIC_90_ values slightly higher but following a similar trend to MIC_50_. This indicates a significant antibiofilm potential of AAEO, consistent with its moderate antimicrobial activity observed in planktonic cultures.

MALDI‐TOF MS analysis of *S. enterica* in the early biofilm growth stage (3 days) showed that the mass spectral profiles of biofilms formed on plastic and glass surfaces and of planktonic cells were very similar, without marked differences in peak presence or relative intensities. This suggests that in the initial phase of biofilm development, the microorganism does not yet display pronounced surface‐dependent spectral changes detectable by MALDI‐TOF MS (Paula et al. 2020). Such findings align with the concept of biofilms as dynamic structures undergoing time‐dependent stages of differentiation (Lambert et al. 2014).

After 7 and 14 days of incubation, more distinct differences in MALDI‐TOF MS spectral patterns were observed. Biofilms on plastic surfaces showed a clearer simplification of spectra and lower overall signal intensities compared to biofilms on glass and planktonic cells. These observations indicate that biofilms developing on different surfaces can acquire distinct spectral characteristics over time, which may reflect surface‐related differences in biofilm organization and physiological state (Gonzalez and Aranda 2023), even though individual proteins or pathways cannot be resolved by this approach.

From a biological perspective, the simplification of MALDI‐TOF MS spectra and the decrease in overall signal intensity observed particularly in biofilms on plastic are consistent with a reduction in biofilm biomass and a shift towards a more stressed or less metabolically active cell population. The dominant signals in the 2–20 kDa range mainly originate from abundant ribosome‐associated and housekeeping proteins, and global changes in these spectral patterns have previously been associated with differences in growth phase and physiological state of bacterial cells (Lambert et al. 2014; Paula et al. 2020). Thus, the surface‐ and time‐dependent spectral changes observed in our study likely reflect structural and physiological remodeling of the biofilm community rather than simple experimental variability.

Following AAEO treatment, several spectra showed a reduction in the intensity of selected peaks or changes in the overall pattern, indicating that exposure to the EO can modify MALDI‐TOF MS profiles of *S. enterica* biofilms. Although MALDI‐TOF Biotyper does not allow detailed quantitative proteomic analysis, these changes are consistent with the hypothesis that AAEO interferes with biofilm structure and cellular integrity, in line with known mechanisms of EOs such as membrane disruption and interference with biofilm‐related functions (Nazzaro et al. 2017). This ability has practical importance for the prevention and control of bacterial biofilms on food‐contact surfaces, which are often sources of persistent contamination and health risks (Donlan 2002).

Cluster analysis of MALDI‐TOF MS spectra confirmed that surface type and incubation time influenced the grouping of biofilm samples, with spectra from plastic and glass biofilms forming separate clusters and later time points diverging from earlier ones. In contrast, planktonic cell spectra from the different sampling times clustered closely together, indicating relatively stable spectral profiles over the incubation period (Hall‐Stoodley et al. 2004). These findings support the view that the observed spectral changes are associated mainly with biofilm growth and surface conditions, rather than with planktonic culture alone.

In summary, our results highlight the complexity of *S. enterica* biofilm adaptations to different surfaces and demonstrate the effective antibiofilm activity of AAEO. To further understand the mechanisms and optimize EO applications in biofilm prevention in food and healthcare sectors, we recommend additional studies focusing on detailed molecular analyses and evaluating combinations of EOs with other preservatives. Although the present study demonstrates a clear inhibitory effect of AAEO on biofilm formation and shows changes in MALDI‐TOF MS spectra of *S. enterica* biofilms after treatment, the underlying molecular mechanisms were not directly investigated. Based on studies of other EOs, membrane disruption, increased permeability, and interference with quorum sensing have been proposed as important mechanisms contributing to biofilm inhibition; however, whether these mechanisms are involved in the action of AAEO remains to be confirmed. Future work should therefore focus on targeted mechanistic assays (e.g., membrane integrity, gene expression related to biofilm regulation) to elucidate how AAEO affects biofilm‐forming cells at the molecular level.

Our results confirmed that the insecticidal activity of AAEO is strongly concentration‐dependent, with efficacy significantly decreasing below the threshold concentration of 25%. This concentration represents the minimum effective dose to achieve a notable mortality effect on *C. maculatus* . The sharp decline in activity at lower concentrations highlights the need for proper dosing for practical applications.

These findings align with literature confirming that the insecticidal effects of EOs are often concentration‐dependent and influenced by specific bioactive components (Isman 2006; Pavela 2015). Additionally, similar concentration‐dependent insecticidal activity of AAEO has been reported against mosquito vectors such as *Anopheles sinensis* (Luo et al. 2022). The mechanism of AAEO action likely involves neurotoxic and physiological disruption of the insect. From a practical perspective, these results suggest that AAEO can be an effective natural alternative in integrated pest management, but careful concentration adjustment and possibly combining with other methods is necessary to maximize efficacy while minimizing environmental impact. In this study, the mode of action of AAEO against *C. maculatus* was not analyzed in detail. Previous work on EOs and other *Artemisia* species suggests that neurotoxic effects, including interference with acetylcholinesterase activity and disruption of the insect nervous system, may play an important role; however, these mechanisms have not yet been demonstrated specifically for AAEO and *C. maculatus* . Targeted mechanistic studies, such as measurements of acetylcholinesterase inhibition or behavioral assays, will be necessary to clarify the molecular basis of the observed insecticidal effects.

Our study comprehensively confirmed the multifunctional biological activities of AAEO, including antimicrobial activity against various Gram‐positive and Gram‐negative bacteria, antibiofilm efficacy against *S. enterica* , and insecticidal activity against the pest *C. maculatus* . In vitro tests demonstrated stronger efficacy of AAEO against Gram‐positive bacteria, while in situ analyses on different food matrices revealed that efficacy depends on the type of matrix and concentration, highlighting the need for tailored application conditions for practical use.

Antibiofilm studies using MALDI‐TOF MS showed time‐ and surface‐dependent changes in spectral profiles of *S. enterica* and indicated that AAEO treatment is associated with modifications of these spectra in biofilms, supporting its potential as an effective natural antibiofilm agent. The insecticidal activity of AAEO was strongly concentration‐dependent, with notable effects observed at concentrations above 25%, which is important for optimal dosing in practical applications.

These findings support the potential of AAEO as a natural and multifunctional bioactive agent applicable in food preservation and integrated pest management. Future research should focus on detailed elucidation of mechanisms of action, optimization of application forms, and evaluation of safety and sensory aspects to maximize the benefits of AAEO in real‐world settings.

This study provides several new insights into the bioactivity of AAEO. First, our in situ vapor‐phase experiments on fresh food matrices offer important information on how the effectiveness of the EO changes under real conditions and across different types of foods. Such tests have been only minimally explored for AAEO so far, and our results demonstrate clear matrix‐dependent differences in antimicrobial activity. Second, the antibiofilm effect of AAEO, for which published data on AAEO are very limited, was evaluated using a combination of crystal violet staining and MALDI‐TOF MS analysis of biofilm samples. This represents a less conventional but useful approach for comparing overall changes in MALDI‐TOF MS spectra of biofilms after EO treatment. To our knowledge, this type of MALDI‐TOF MS application has not been reported for AAEO. Third, the results of the fumigant insecticidal assays provide new data on the susceptibility of the storage pest *C. maculatus* to AAEO. Although insecticidal effects of EOs have been described for other *Artemisia* species, available information for AAEO and this particular stored‐product pest is very limited. These three aspects constitute new contributions that extend current knowledge of AAEO and highlight its potential applications in food preservation and stored‐product protection.

## 5 Conclusion

The study confirmed that AAEO possesses multifunctional biological activity with significant potential for applications in food preservation, post‐harvest crop protection, and integrated pest management. Chemical analysis revealed that the oil is rich in bioactive compounds, primarily 1,8‐cineole, β‐caryophyllene, and longifolene, which likely contribute to its efficacy. In vitro tests demonstrated mild to moderate antimicrobial activity, with stronger effects against Gram‐positive bacteria, while in situ tests on various food matrices showed that the oil's effectiveness depends on the matrix type and concentration, highlighting the need for tailored application. Antibiofilm assays and MALDI‐TOF MS analyses showed AAEO's ability to interfere with biofilm formation by *Salmonella enterica* and to modify its MALDI‐TOF MS spectral profiles, underscoring the oil's potential in preventing and controlling biofilm‐related contamination on food‐contact surfaces. Insecticidal tests against *C. maculatus* revealed strong activity at concentrations above 25%, achieving 100% mortality at 100% concentration, indicating its suitability as a natural insecticide in storage facilities. Despite these positive outcomes, further research is necessary to elucidate the mechanisms of AAEO action, optimize its applications, and evaluate safety and sensory aspects to fully exploit its potential under practical conditions. Overall, this study contributes to the development of natural, effective, and sustainable solutions in food safety, post‐harvest crop protection, and integrated pest management.

## Author Contributions

**Miroslava Kačániová:** methodology, investigation, writing‐original draft, formal analyses, supervision, project administration, funding acquisition, visualization. **Zhaojun Ban:** formal analyses, writing‐review and editing. **Li Li:** formal analyses, writing‐review and editing. **Jian Lou:** formal analyses, writing‐review and editing. **Joel Horacio Elizondo‐Luevano:** formal analyses, writing‐review and editing. **Anis Ben Hsouna:** formal analyses, Writing‐review and editing. **Rania Ben Saad:** formal analyses, writing‐review and editing. **Alessandro Bianchi:** statistics, formal analyses, methodology, writing‐original draft. **Ladislav Bakay:** formal analyses, writing‐review and editing. **Stefania Garzoli:** formal analyses, data curation, writing‐review and editing, supervision.

## Funding

This research was funded by the grant APVV‐20‐0058 “The potential of the essential oils from aromatic plants for medical use and food preservation and by the VEGA” grant 1/0059/24 titled “Chemical properties and biological activity (in vitro, in vivo and in situ) of plant volatile mixtures, their main components and inclusion systems”.

## Conflicts of Interest

The authors declare no conflicts of interest.

## Data Availability

The authors have nothing to report.

## References

[fsn371385-bib-0001] Aćimović, M. , J. S. Jeremić , M. Todosijević , et al. 2022. “Comparative Study of the Essential Oil and Hydrosol Composition of Sweet Wormwood ( *Artemisia annua* L.) From Serbia.” Chemistry & Biodiversity 19: e202100954. 10.1002/cbdv.202100954. 35170197

[fsn371385-bib-0002] Akbas, M. Y. , and T. Kokumer . 2015. “The Prevention and Removal of Biofilm Formation of *Staphylococcus aureus* Strains Isolated From Raw Milk Samples by Citric Acid Treatments.” International Journal of Food Science and Technology 50: 1666–1672. 10.1111/ijfs.12823.

[fsn371385-bib-0003] Ali, E. A. , D. M. Mohammed , F. A. El Gawad , M. A. Orabi , R. K. Gupta , and P. P. Srivastav . 2025. “Valorization of Food Processing Waste Byproducts for Essential Oil Production and Their Application in Food System.” Waste Management Bulletin 3: 100200. 10.1016/j.wmb.2025.100200.

[fsn371385-bib-0004] Burt, S. 2004. “Essential Oils: Their Antibacterial Properties and Potential Applications in Foods—A Review.” International Journal of Food Microbiology 94: 223–253. 10.1016/j.ijfoodmicro.2004.03.022. 15246235

[fsn371385-bib-0005] Cai, Z.‐M. , J.‐Q. Peng , Y. Chen , et al. 2021. “1,8‐Cineole: A Review of Source, Biological Activities, and Application.” Journal of Asian Natural Products Research 23: 938–954. 10.1080/10286020.2020.1839432. 33111547

[fsn371385-bib-0006] Caputo, P. , M. C. Di Martino , B. Perfetto , F. Iovino , and G. Donnarumma . 2018. “Use of MALDI‐TOF MS to Discriminate Between Biofilm‐Producer and Non‐Producer Strains of *Staphylococcus epidermidis* .” International Journal of Environmental Research and Public Health 15: 1695. 10.3390/ijerph15081695. 30096872 PMC6121576

[fsn371385-bib-0007] Carrascosa, C. , D. Raheem , F. Ramos , A. Saraiva , and A. Raposo . 2021. “Microbial Biofilms in the Food Industry—A Comprehensive Review.” International Journal of Environmental Research and Public Health 18: 2014. 10.3390/ijerph18042014. 33669645 PMC7922197

[fsn371385-bib-0008] Chauhan, N. , U. Kashyap , S. K. Dolma , and S. G. E. Reddy . 2022. “Chemical Composition, Insecticidal, Persistence and Detoxification Enzyme Inhibition Activities of Essential Oil of *Artemisia maritima* Against the Pulse Beetle.” Molecules 27: 1547. 10.3390/molecules27051547. 35268647 PMC8911588

[fsn371385-bib-0009] Chen, L.‐L. , H.‐J. Zhang , J. Chao , and J.‐F. Liu . 2017. “Essential Oil of *Artemisia argyi* Suppresses Inflammatory Responses by Inhibiting JAK/STATs Activation.” Journal of Ethnopharmacology 204: 107–117. 10.1016/j.jep.2017.04.017. 28438564

[fsn371385-bib-0010] Chizzola, R. , F. Billiani , S. Singer , and J. Novak . 2021. “Diversity of Essential Oils and the Respective Hydrolates Obtained From Three *Pinus cembra* Populations in the Austrian Alps.” Applied Sciences 11: 5686. 10.3390/app11125686.

[fsn371385-bib-0011] Danh, L. T. , L. N. Han , N. D. A. Triet , J. Zhao , R. Mammucari , and N. Foster . 2013. “Comparison of Chemical Composition, Antioxidant and Antimicrobial Activity of Lavender (*Lavandula angustifolia* L.) Essential Oils Extracted by Supercritical CO2, Hexane and Hydrodistillation.” Food and Bioprocess Technology 6: 3481–3489. 10.1007/s11947-012-1026-z.

[fsn371385-bib-0012] Diao, W.‐R. , L.‐L. Zhang , S.‐S. Feng , and J.‐G. Xu . 2014. “Chemical Composition, Antibacterial Activity, and Mechanism of Action of the Essential Oil From *Amomum kravanh* .” Journal of Food Protection 77: 1740–1746. 10.4315/0362-028x.jfp-14-014. 25285491

[fsn371385-bib-0013] Donlan, R. M. 2002. “Biofilms: Microbial Life on Surfaces.” Emerging Infectious Diseases 8: 881–890. 10.3201/eid0809.020063. 12194761 PMC2732559

[fsn371385-bib-0014] Ehrnhöfer‐Ressler, M. M. , K. Fricke , M. Pignitter , et al. 2013. “Identification of 1,8‐Cineole, Borneol, Camphor, and Thujone as Anti‐Inflammatory Compounds in a *Salvia officinalis* L. Infusion Using Human Gingival Fibroblasts.” Journal of Agricultural and Food Chemistry 61: 3451–3459. 10.1021/jf305472t. 23488631

[fsn371385-bib-0015] Flores‐Flores, A. S. , J. M. Vazquez‐Guillen , P. Bocanegra‐Ibarias , et al. 2025. “MALDI‐TOF MS Profiling to Predict Resistance or Biofilm Production in Gram‐Positive ESKAPE Pathogens From Healthcare‐Associated Infections.” Diagnostic Microbiology and Infectious Disease 111: 116562. 10.1016/j.diagmicrobio.2024.116562. 39426117

[fsn371385-bib-0016] Gonzalez, J. M. , and B. Aranda . 2023. “Microbial Growth Under Limiting Conditions‐Future Perspectives.” Microorganisms 11: 1641. 10.3390/microorganisms11071641. 37512814 PMC10383181

[fsn371385-bib-0017] Guan, X. , D. Ge , S. Li , K. Huang , J. Liu , and F. Li . 2019. “Chemical Composition and Antimicrobial Activities of *Artemisia argyi* Lévl. Et Vant Essential Oils Extracted by Simultaneous Distillation‐Extraction, Subcritical Extraction and Hydrodistillation.” Molecules 24: 483. 10.3390/molecules24030483. 30700013 PMC6384757

[fsn371385-bib-0018] Hall‐Stoodley, L. , J. W. Costerton , and P. Stoodley . 2004. “Bacterial Biofilms: From the Natural Environment to Infectious Diseases.” Nature Reviews. Microbiology 2: 95–108. 10.1038/nrmicro821. 15040259

[fsn371385-bib-0019] Heo, S. , and S. Chung . 2023. “Development of Phenomic Approaches to Estimate Harvesting Time of *Artemisia argyi* and Classifying Its Quality Based on Flavonoids, Essential Oil and Leaf Weight Ratio.” Plant Biotechnology Reports 17: 459–469. 10.1007/s11816-023-00846-5.

[fsn371385-bib-0020] Hong, M. , M. Kim , H. Jang , et al. 2023. “Multivariate Analysis of Essential Oil Composition of *Artemisia annua* L. Collected From Different Locations in Korea.” Molecules 28: 1131. 10.3390/molecules28031131. 36770797 PMC9920137

[fsn371385-bib-0021] Huang, H.‐C. , H.‐F. Wang , K.‐H. Yih , L.‐Z. Chang , and T.‐M. Chang . 2012. “Dual Bioactivities of Essential Oil Extracted From the Leaves of *Artemisia argyi* as an Antimelanogenic Versus Antioxidant Agent and Chemical Composition Analysis by GC/MS.” IJMS 13: 14679–14697. 10.3390/ijms131114679. 23203088 PMC3509604

[fsn371385-bib-0022] Hwang, H.‐J. , D. Li , J. Lee , M. K. Kang , H. R. Moon , and J.‐H. Lee . 2023. “Compounds That Have an Anti‐Biofilm Effect Against Common Bacteria at Very Low Concentrations and Their Antibiotic Combination Effect.” Antibiotics 12: 853. 10.3390/antibiotics12050853. 37237757 PMC10215624

[fsn371385-bib-0023] Hyldgaard, M. , T. Mygind , and R. L. Meyer . 2012. “Essential Oils in Food Preservation: Mode of Action, Synergies, and Interactions With Food Matrix Components.” Frontiers in Microbiology 3: 12. 10.3389/fmicb.2012.00012. 22291693 PMC3265747

[fsn371385-bib-0024] Isman, M. B. 2006. “Botanical Insecticides, Deterrents, and Repellents in Modern Agriculture and an Increasingly Regulated World.” Annual Review of Entomology 51: 45–66. 10.1146/annurev.ento.51.110104.151146. 16332203

[fsn371385-bib-0025] Kačániová, M. , L. Galovičová , E. Ivanišová , et al. 2020. “Antioxidant, Antimicrobial and Antibiofilm Activity of Coriander (*Coriandrum sativum* L.) Essential Oil for Its Application in Foods.” Food 9: 282. 10.3390/foods9030282. PMC7142854 32143314

[fsn371385-bib-0026] Kačániová, M. , L. Galovičová , V. Valková , et al. 2021. “Chemical Composition and Biological Activity of *Salvia officinalis* Essential Oil.” Acta Horticulturae et Regiotecturae 24: 81–88. 10.2478/ahr-2021-0028.

[fsn371385-bib-0027] Kačániová, M. , N. L. Vukovic , N. Čmiková , et al. 2023. “ *Salvia sclarea* Essential Oil Chemical Composition and Biological Activities.” International Journal of Molecular Sciences 24: 5179. 10.3390/ijms24065179. 36982252 PMC10049179

[fsn371385-bib-0028] Kamal, G. M. , J. Uddin , M. Asmari , et al. 2025. “Natural Polyphenols as a Promising Aquatic Food Preservative: A Concurrent Review.” Journal of Agriculture and Food Research 22: 102046. 10.1016/j.jafr.2025.102046.

[fsn371385-bib-0029] Khan, S. , A. Abdo , Y. Shu , Z. Zhang , and T. Liang . 2023. “The Extraction and Impact of Essential Oils on Bioactive Films and Food Preservation, With Emphasis on Antioxidant and Antibacterial Activities—A Review.” Food 12: 4169. 10.3390/foods12224169. PMC10670266 38002226

[fsn371385-bib-0030] Kiran, S. , and B. Prakash . 2015. “Assessment of Toxicity, Antifeedant Activity, and Biochemical Responses in Stored‐Grain Insects Exposed to Lethal and Sublethal Doses of *Gaultheria procumbens* L. Essential Oil.” Journal of Agricultural and Food Chemistry 63: 10518–10524. 10.1021/acs.jafc.5b03797. 26558484

[fsn371385-bib-0031] Lambert, G. , A. Bergman , Q. Zhang , D. Bortz , and R. Austin . 2014. “Physics of Biofilms: The Initial Stages of Biofilm Formation and Dynamics.” New Journal of Physics 16: 045005. 10.1088/1367-2630/16/4/045005.

[fsn371385-bib-0032] Li, D. , R. Wang , M. You , N. Chen , L. Sun , and N. Chen . 2024. “The Antimicrobial Effect and Mechanism of the *Artemisia argyi* Essential Oil Against Bacteria and Fungus.” Brazilian Journal of Microbiology 55: 727–735. 10.1007/s42770-023-01172-2. 37957442 PMC10920523

[fsn371385-bib-0033] Li, J. , H. Chen , C. Guo , et al. 2023. “ *Artemisia argyi* Essential Oil Exerts Herbicidal Activity by Inhibiting Photosynthesis and Causing Oxidative Damage.” Industrial Crops and Products 194: 116258. 10.1016/j.indcrop.2023.116258.

[fsn371385-bib-0034] Liu, Y. , Y. He , F. Wang , et al. 2021. “From Longevity Grass to Contemporary Soft Gold: Explore the Chemical Constituents, Pharmacology, and Toxicology of *Artemisia argyi* H. Lév. & Vaniot Essential Oil.” Journal of Ethnopharmacology 279: 114404. 10.1016/j.jep.2021.114404. 34246739

[fsn371385-bib-0035] Luo, D.‐Y. , Z.‐T. Yan , L.‐R. Che , J. J. Zhu , and B. Chen . 2022. “Repellency and Insecticidal Activity of Seven Mugwort (*Artemisia argyi*) Essential Oils Against the Malaria Vector Anopheles Sinensis.” Scientific Reports 12: 5337. 10.1038/s41598-022-09190-0. 35351963 PMC8964668

[fsn371385-bib-0036] Nazzaro, F. , F. Fratianni , R. Coppola , and V. D. Feo . 2017. “Essential Oils and Antifungal Activity.” Pharmaceuticals 10: 86. 10.3390/ph10040086. 29099084 PMC5748643

[fsn371385-bib-0037] Ozek, G. , Y. Suleimen , N. Tabanca , et al. 2014. “Chemical Diversity and Biological Activity of the Volatiles of Five Artemisia Species From Far East Russia.” Records of Natural Products 8: 242–261.

[fsn371385-bib-0038] Palomares‐Navarro, J. J. , A. T. Bernal‐Mercado , G. A. González‐Aguilar , L. A. Ortega‐Ramirez , M. A. Martínez‐Téllez , and J. F. Ayala‐Zavala . 2022. “Antibiofilm Action of Plant Terpenes in *Salmonella* Strains: Potential Inhibitors of the Synthesis of Extracellular Polymeric Substances.” Pathogens 12: 35. 10.3390/pathogens12010035. 36678383 PMC9864247

[fsn371385-bib-0039] Paula, A. J. , G. Hwang , and H. Koo . 2020. “Dynamics of Bacterial Population Growth in Biofilms Resemble Spatial and Structural Aspects of Urbanization.” Nature Communications 11: 1354. 10.1038/s41467-020-15165-4. PMC7070081 32170131

[fsn371385-bib-0040] Pavela, R. 2015. “Essential Oils for the Development of Eco‐Friendly Mosquito Larvicides: A Review.” Industrial Crops and Products 76: 174–187. 10.1016/j.indcrop.2015.06.050.

[fsn371385-bib-0041] Pedreira, A. , N. Martínez‐López , J. A. Vázquez , and M. R. García . 2024. “Modelling the Antimicrobial Effect of Food Preservatives in Bacteria: Application to *Escherichia coli* and *Bacillus cereus* Inhibition With Carvacrol.” Journal of Food Engineering 361: 111734. 10.1016/j.jfoodeng.2023.111734.

[fsn371385-bib-0042] Roy, R. , M. Tiwari , G. Donelli , and V. Tiwari . 2018. “Strategies for Combating Bacterial Biofilms: A Focus on Anti‐Biofilm Agents and Their Mechanisms of Action.” Virulence 9: 522–554. 10.1080/21505594.2017.1313372. 28362216 PMC5955472

[fsn371385-bib-0043] Sánchez‐González, L. , A. Chiralt , C. González‐Martínez , and M. Cháfer . 2011. “Effect of Essential Oils on Properties of Film Forming Emulsions and Films Based on Hydroxypropylmethylcellulose and Chitosan.” Journal of Food Engineering 105: 246–253. 10.1016/j.jfoodeng.2011.02.028.

[fsn371385-bib-0044] Shan, J. , and H. Zhou . 2021. “Chemical Constituents, Antibacterial and Coagulation Activity of the Essential Oil From the Stem of *Artemisia argyle* H. Lév.” American Journal of Biochemistry and Biotechnology 17: 241–247. 10.3844/ajbbsp.2021.241.247.

[fsn371385-bib-0045] Srivastava, N. , B. Singh , D. Chanda , and K. Shanker . 2015. “Chemical Composition and Acetylcholinesterase Inhibitory Activity of *Artemisia maderaspatana* Essential Oil.” Pharmaceutical Biology 53: 1677–1683. 10.3109/13880209.2014.1001405. 25885940

[fsn371385-bib-0046] Tomi, K. , M. Kitao , N. Konishi , H. Murakami , Y. Matsumura , and T. Hayashi . 2016. “Enantioselective GC–MS Analysis of Volatile Components From Rosemary ( *Rosmarinus officinalis* L.) Essential Oils and Hydrosols.” Bioscience, Biotechnology, and Biochemistry 80: 840–847. 10.1080/09168451.2016.1146066. 26923429

[fsn371385-bib-0047] Touati, A. , A. Mairi , N. A. Ibrahim , and T. Idres . 2025. “Essential Oils for Biofilm Control: Mechanisms, Synergies, and Translational Challenges in the Era of Antimicrobial Resistance.” Antibiotics 14: 503. 10.3390/antibiotics14050503. 40426569 PMC12108346

[fsn371385-bib-0048] Tsai, Y.‐H. , and P. J. Lein . 2021. “Mechanisms of Organophosphate Neurotoxicity.” Current Opinion in Toxicology 26: 49–60. 10.1016/j.cotox.2021.04.002. 34308007 PMC8302047

[fsn371385-bib-0049] Wang, W. , X. Zhang , N. Wu , Y. Fu , and Y. Zu . 2006. “Antimicrobial Activities of Essential Oil From *Artemisiae argyi* Leaves.” Journal of Forestry Research 17: 332–334. 10.1007/s11676-006-0077-2.

[fsn371385-bib-0050] Wen, W. , H. Xiang , H. Qiu , et al. 2024. “Screening and Identification of Antibacterial Components in in This Regard, I'd Like to Ask You if You Have Additional Results From Tests With Hydrolates. I've Completed the Chemical Analyses and Can Send You the Compositional Data if You Think They Might Be Useful for Writing the Papers. Essential Oil by TLC–Direct Bioautography Combined With Comprehensive 2D GC × GC‐TOFMS.” Journal of Chromatography B 1234: 124026. 10.1016/j.jchromb.2024.124026. 38277723

[fsn371385-bib-0051] Wenqiang, G. , L. Shufen , Y. Ruixiang , and H. Yanfeng . 2006. “Comparison of Composition and Antifungal Activity of *Artemisia argyi Lévl. et Vant* Inflorescence Essential Oil Extracted by Hydrodistillation and Supercritical Carbon Dioxide.” Natural Product Research 20: 992–998. 10.1080/14786410600921599. 17032625

[fsn371385-bib-0052] Xiang, F. , J. Bai , X. Tan , T. Chen , W. Yang , and F. He . 2018. “Antimicrobial Activities and Mechanism of the Essential Oil From *Artemisia argyi* Levl. et Van. Var. Argyi cv. Qiai.” Industrial Crops and Products 125: 582–587. 10.1016/j.indcrop.2018.09.048.

[fsn371385-bib-0053] Xu, Z. , Y. Liang , S. Lin , et al. 2016. “Crystal Violet and XTT Assays on *Staphylococcus aureus* Biofilm Quantification.” Current Microbiology 73: 474–482. 10.1007/s00284-016-1081-1. 27324342

[fsn371385-bib-0054] Yaron, S. , and U. Römling . 2014. “Biofilm Formation by Enteric Pathogens and Its Role in Plant Colonization and Persistence.” Microbial Biotechnology 7: 496–516. 10.1111/1751-7915.12186. 25351039 PMC4265070

[fsn371385-bib-0055] Zhang, W.‐J. , C.‐X. You , K. Yang , et al. 2014. “Bioactivity of Essential Oil of *Artemisia argyi* Lévl. Et Van. And Its Main Compounds Against *Lasioderma serricorne* .” Journal of Oleo Science 63: 829–837. 10.5650/jos.ess14057. 25017866

